# Impact of Home-Based Learning Experience During COVID-19 on Future Intentions to Study Online: A Chinese University Perspective

**DOI:** 10.3389/fpsyg.2022.862965

**Published:** 2022-03-23

**Authors:** Liang Zhao, Yibin Ao, Yan Wang, Tong Wang

**Affiliations:** ^1^Office of Academic Affairs, Chengdu University of Technology, Chengdu, China; ^2^College of Environment and Civil Engineering, Chengdu University of Technology, Chengdu, China; ^3^Department of Engineering Management, Sichuan College of Architectural Technology, Chengdu, China; ^4^Faculty of Architecture and the Built Environment, Delft University of Technology, Delft, Netherlands

**Keywords:** online learning, home-based learning experience, flow experience theory, theory of planned behavior, COVID-19, blended learning design

## Abstract

As coronavirus disease 2019 (COVID-19) swept the world in early 2020, all the Chinese universities and colleges adopted online learning to fulfill the directive saying “classes suspended but learning continues.” Understanding the impact of this large-scale online learning experience on the future online learning intention of Chinese university students can help design better blended-learning activities. This study applies flow experience and theory of planned behavior (TPB) to construct a theoretical framework for assumption making and the assumptions made are validated by data gained from questionnaires. A total of 6,933 students from 54 institutions in China participated in the investigation, with 5,456 valid questionnaires returned. This study employs partial least squares (PLS) regression and confirmative factor analysis (CFA) to analyze and estimate the measurement model and the structural model. The results indicate that the experience of home-based learning significantly influenced the attitudes of Chinese university students, which in turn had a positive influence on their intention to continue online learning. The research findings provide a theoretical framework and practical guidelines on building a scientific online learning platform with appropriate online learning environments and tasks for a post-COVID-19 era blended-learning in Chinese universities.

## Introduction

During coronavirus disease 2019 (COVID-19) pandemic, many countries suspended classes worldwide, affecting over 1.7 billion students, according to United Nations Educational, Scientific and Cultural Organization (UNESCO) statistics. In China, the Ministry of Education issued a Notice on the Postponement of the Spring Semester on January 27th, 2020, and the State Council held a press conference on the joint prevention and control mechanism and issued a directive, “classes suspended but learning continues” on February 12th, 2020, requiring universities and colleges in China to conduct online education. According to statistics, as of May 8th, 1,454 colleges and universities across the country were engaged in online education, with 1.03 million teachers offering 1.07 million courses, a total of 12.26 million course sessions for both the theoretical and experimental contents. In total, 17.75 million university students participated in 2.3 billion sessions, making online education the main mode of education in China’s colleges and universities during COVID-19 pandemic (MOE, 2020).

Online learning is a form of learning that requires a high degree of independence and autonomy from students and is blended with traditional face-to-face classroom instruction to form a blended learning model. Online learning not only changes the existing curriculum but also creates a new form of teaching and learning (Bonk and Graham, 2005). The online teaching model, based on modern information technology, is beneficial for expanding teaching scale, increasing teaching efficiency, and improving learning outcomes, but it also creates many new problems (Wang et al., 2020). Research indicates that although online learning models give learners a high degree of autonomy, their motivation to continue learning remains low (Kang et al., 2014). The virtuality of the medium makes learners feel disconnected from the real world, resulting in feelings of isolation and reduced learning engagement (Rovai, 2003; Waugh and Su-Searle, 2014). In addition, online learning lacks face-to-face communication, making it difficult to access timely guidance and attention (Guo et al., 2016).

While past online learning experiences were characterized by high self-selection and large differences in individual learning environments (Cao, 2014), online learning during COVID-19 pandemic had two new features: on the one hand, it was highly mandatory and prescriptive as an alternative to offline learning during the pandemic and was consistent with the planned offline classroom in terms of learning contents. On the other hand, online teaching during the pandemic was the first large-scale experiment on a global scale. According to Yan Wu, Director General of the Department of Higher Education of the Ministry of Education of China, “Higher education can no longer and should not revert to the state of teaching and learning before the pandemic, and online education will become the new normal in the future” (MOE, 2020). Therefore, exploring the impact of the large-scale college students’ online learning experience during the epidemic on their willingness to continue online learning has important theoretical and practical implications for the further design of the blended learning model of higher education.

The article is structured as follows: Section “Literature Review” presents a detailed literature review. Section “Research framework and hypothesis” illustrates the research framework and the research hypothesis. Section “Research Methods” explains the research design, followed by data analysis results in Section “Data Analysis Results.” Conclusions are presented in Section “Conclusion.”

## Literature Review

### Flow Experience Theory in Online Learning

The concept of flow experience was first proposed by psychologist Csikszentmihalyi in the 1970s and 1980s. It refers to the overall feeling people get when they are fully engaged in an activity; i.e., when people are fully absorbed in an activity, they often forget the passage of time and the perception of things around them, or even lose their sense of self-awareness, and feel a constant sense of pleasure (Csikszentmihalyi and LeFevre, 1989). Originally applied to the field of psychology, this theory has been utilized in many different studies, such as those on games, dance, rock climbing, shopping, and sports (Lu et al., 2009). It has now been introduced into the area of online learning (Lee and Choi, 2013), to study the impact of network user experience on usage intention and behavior.

The flow experience model, constructed by a process-driven approach, has been widely used in empirical studies of online learning. For instance, Davis and Wong (2007), who combined the flow experience and technology acceptance model (TAM) theory, discovered that the acquisition of the necessary skills to overcome course-related challenges is critical to students’ ability to generate a flow experience and to continue learning. Shin (2006) verified that the balance of skills and challenges for online learners could significantly affect their flow experience and found that the flow experience greatly affected students’ satisfaction with online learning. The many factors that contribute to the user’s flow experience can be grouped into three dimensions, person (P), artifact (A), and task (T), according to the person-artifact-task model (PAT model) proposed by Finneran and Zhang (2003). The artifact restricts the possibility of the user’s access to new media, while the user’s characteristics and state determine the experience of each use of the product. Only when users have clear task goals and are proficient with the artifact can they generate mental flow and enter a state of ecstasy (Chen et al., 2019).

### Behavioral Science in the Online Learning Experience

Existing research shows that learners’ experiences in online environments are influenced by a variety of factors (Liu and Zhang, 2017), and online learning experiences largely influence learners’ intentions and behaviors to continue online learning (Davis and Wong, 2007; Lee, 2010). The online learning environment, online learning resources, teacher–student and peer interactions (Paechter et al., 2010), collaborative learning (Sakulwichitsintu et al., 2015), course tasks, and the design of course activities (Jiang et al., 2019) are considered to be important factors influencing online learning experiences. To predict learning behaviors and explore directions and paths for improvement, researchers have applied influence factor modeling to screen and identify influences on learning behaviors (Alraimi et al., 2015). Behavioral science theory is often used to construct online learning influence factor models and to study human behavior, mainly from the perspective of human needs, desires, motivations, purposes, and other psychological aspects (Liu and Sun, 2005).

Behavioral science is mainly based on theories such as the theory of rational behavior (TRA), theory of planned behavior (TPB), TAM, and expectation confirmation model (ECM-ISC), among others. Different behavioral science theories carry different assumptions about the environment in which learning behavior occurs. For example, TAM and ECM-ISC are only applicable regarding the willingness of adopting information systems or online learning systems, while TRA and TPB apply to a wider range of online and blended learning environments (Kong et al., 2017).

The TRA was originally proposed by American scholars (Fishbein and Ajzen, 1975). In the TRA, behavioral intention is the key factor that causes the individual to perform a certain behavior, which can be understood as jointly determined by the subjective norm and the individual’s attitude toward the behavior to be performed. Attitude is a predictor of behavioral intentions and is an individual’s overall evaluation of the behavior (Conner and Armitage, 1998). Specifically, in the study of online learning behavior, “attitude” can be understood as the predisposition, positive or negative, that learners hold toward online learning behavior, which is determined by the individual’s beliefs about the outcome of the behavior and their assessment of its importance (Kong et al., 2017). The basic assumption of the TRA is that most human behavior is rational and self-controllable and therefore is weak in explaining factors influencing uncontrollable behavior.

Accordingly, Ajzen (1991) extended the TRA by adding a third determinant, namely, perceived behavioral control, and proposed the TPB. Perceived behavioral control is defined as behavior in which beliefs are related to whether a person can successfully obtain the necessary resources and opportunities to perform the completed behavior, weighted by the perceived ability of each factor to facilitate or inhibit the behavior. Perceptions of factors that may facilitate or inhibit behavioral performance are referred to as control beliefs. These factors include internal control factors (information, skills, emotions, etc.) and external control factors (opportunity, dependence on others, barriers, etc.). The new theory constructed by introducing the concept of perceived behavioral control goes beyond the main hypothesis proposed by TRA regarding the complete control of behavior by personal willpower (Sheppard et al., 1988). TPB has been found in different empirical studies to significantly improve the explanatory power of behavior (Ajzen, 1991). In this model, attitudes, subjective norms, and perceived behavioral control are the three main determinants of behavioral intentions. The theory assumes that the more positive the attitude, the greater the subjective norm, and the stronger the perceived behavioral control, the stronger the behavioral willingness and the more likely the actual behavior will occur.

### Combination of Theories

Researchers tend to draw on other disciplines and well-established theories to guide their research so that they not only develop and test more comprehensive and integrated models to fill in missing causal relationships but also use other well-established research to supplement their arguments and theoretical foundations (Karahanna and Straub, 1999). Recent research has shown that either extending the original rational choice model theory or combining it with other social psychological theories can yield better results and explanatory power than simply using the original rational choice model (Han, 2015). A major limitation of TPB theory is considered to be its lack of attention to the drivers of belief-based behavioral intentions (Hagger et al., 2002; Hagger and Chatzisarantis, 2009), so researchers often test and develop more complex models to improve the explanatory power of the theory. Lee (2010), for example, has comprehensively integrated and extended the ECM-ISC, TAM, TPB, and theory of flow experience in his research explaining learners’ willingness to continue using online learning. Several past studies have also validated the rationale for integrating the TPB and the theory of flow experience (Alzahrani et al., 2017).

## Research Framework and Hypothesis

As mentioned in Section “Flow Experience Theory in Online Learning,” online learning experiences largely influence learners’ intentions and behaviors to continue online learning and flow experience is an extremely enjoyable psychological state and temporary subjective experience which is deemed to be an intrinsic motivator in explaining learner’s willingness (Davis and Wong, 2007; Lee, 2010). Therefore, an important prerequisite to predicting future learners’ intention to continue engaging in online learning is the flow experience of large-scale online learning during the pandemic.

As explained in Section “Behavioral Science in the Online Learning Experience,” the TPB has been found in different empirical studies to significantly improve the explanatory power of behavior (Ajzen, 1991) and is considered to be one of the most dominant theories in the field of behavior science (Armitage and Conner, 2001). Many studies have also extended the theory, adapting the theoretical framework to enhance its validity and adequacy. Therefore, this study starts from the theory of flow experience which identifies the experience of large-scale online learning during the pandemic and combines it with the TPB to predict learners’ intention to continue to engage in online learning after the pandemic in the Chinese higher education context.

**FIGURE 1:**
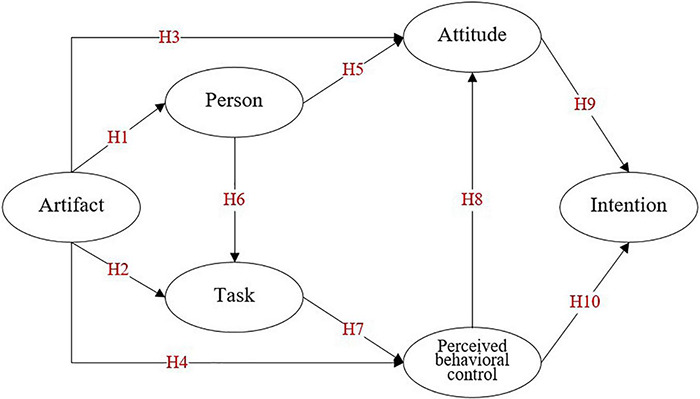
Research framework.

Furthermore, due to the isolation of learners’ home-based learning, the influence of other participants in the teaching session on learners is reduced. Before the formal investigation, we found that the interaction effect between students was really small through network interviews. As such, this study assumes that subjective external pressure (perceptions) has no effect on online learners during the pandemic. With this in mind, this article builds a research framework as shown in Figure 1, taking the perspective of endogenous psychological factors concerning the three dimensions of the PAT model of mental flow (Finneran and Zhang, 2003) and considering primarily the influence on the two elements in the TPB, namely, attitudes and perceived behavioral control, on the students’ intention to further online learning after the pandemic.

During COVID-19 pandemic, Chinese universities have made a large-scale transformation from offline learning to online learning, which placed high demands on the teaching platforms and shaped the characteristics of the learners’ experience of using the platforms during this period. The experience of this artifact is the most intuitive in the shifting process of learning mode. In the field of computer-mediated environments, some studies indicate that users can experience mental flow when using artifacts such as spreadsheets and word processors (Webster et al., 1993; Ghani, 1995). According to Csikszentmihalyi (1990), the experience of mental flow is more typical and pronounced when the user interacts with a toy. For online learning, mental flow is facilitated when the learner’s experience of artifacts and platforms is as good and pleasant as that of toys. In the antecedent conditions that produce flow experiences, artifacts and tasks are closely related. Norman (1988) describes the correlation between them in this way: “When I use a direct manipulation system—whether for text editing, drawing pictures, or creating and playing video games—I do think of myself not as using a computer but at doing the particular task. The computer is, in effect, invisible.” This means that when a good artifact experience is matched with a task challenge, it allows the learner to concentrate on the task when facing the challenge. Therefore, this article proposes the following hypotheses:

**H1:** During the pandemic, learners’ experience with artifacts used in online learning has a significant positive impact on individual learners’ states regarding online learning.
**H2:** During the pandemic, learners’ experience with artifacts used in online learning has a significant positive effect on task challenges undertaken.

According to the PAT model, artifact factors include system quality (e.g., interface usability, perceived ease of use, controllability, and interactivity), information quality, and service quality (Li and Xiao, 2019). For online learning, the artifact experience mainly includes learners’ perceptions of artifact usability and ease of use when using the platform, while ease of use of the system is influenced by the resource form and task characteristics, and system usefulness is influenced by resource content and source (Ji, 2013). Therefore, the artifact experience also includes learners’ perceptions of the richness and quality of online learning platform resources. Among them, perceived usefulness and perceived ease of use are a pair of concepts developed by Davis (1989) based on the TRA to construct a TAM.

The model assumes that perceived usefulness and perceived ease of use have a direct effect on behavioral attitudes and, consequently, indirectly affect behavioral intentions. The TAM has been proposed for the use of information systems, and existing research has found that usefulness and ease of use act as antecedent variables in the generation of mental flow, which in turn affects attitudes, intentions, and usage behavior (Sánchez-Franco, 2007). This theory is also applicable to the study of online learning systems: if users perceive that online learning platforms have better usefulness, ease of use and powerful features, they will advance their learning process to a certain extent, making them more willing to embrace online learning (Yang and Yan, 2011). In addition, it has been found that when users enjoy using artifacts to look for information, they also regard them as easy to use and are willing to use them in the future (Liaw and Huang, 2003). On this basis, this article proposes the following hypotheses:

**H3:** During the pandemic, learners’ experience with artifacts used in online learning has a significant positive impact on their attitude toward continuing online learning after the pandemic.
**H4:** During the pandemic, learners’ experience with artifacts used in online learning has a significant positive effect on their perceived behavioral control.

In the PAT model (Finneran and Zhang, 2003), human factors include skills, motivation (Hoffman and Novak, 1996), curiosity, intrinsic interest (Webster et al., 1993), and other aspects. Characteristics of an individual and psychological states influence the generation of the mental flow experience. Wang et al. (2017) categorized highly focused attention, loss of self-awareness, and time distortion as experiential factors based on the nine dimensions of mental flow experience proposed by Csikszentmihalyi (1997). All of these factors point to the individual state of a person during the mental flow experience. Presence is also a very important individual state in online learning, which is the feeling of being immersed in an environment shaped by a medium and having a sense of reality, or “being personally on the scene” (Kim and Biocca, 1997). The sense of presence in online learning is a feeling like taking a class in a real space, with a strong sense of the presence of the teacher and other students, which reflects the feeling of “being with others.” Social presence helps reduce the sense of loneliness that may appear in the online learning environment, which makes up for this deficiency in online learning (Zhao et al., 2018). It has been shown that presence is an important condition for generating a mental flow experience (Finneran and Zhang, 2005; Nah et al., 2011) and that if learners can enjoy the experience of online learning activities, they will not only engage in online learning but will also be intrinsically motivated to do so by consistently engaging in online learning to acquire these positive feelings (Davis and Wong, 2007). Research shows that increased learning, exploratory, and participatory behaviors, and positive subjective experiences are important outcomes of mental flow (Hoffman and Novak, 1996) and that with the mental flow, learners will devote more time and energy to the learning task, have a more adequate initiative to deal with the challenge, and show significantly enhanced attitudes (Richard and Chandra, 2005; Sánchez-Franco, 2007). Therefore, this article proposes the following hypotheses:

**H5:** During the pandemic, individual learners’ states of online learning have a significant positive effect on their attitude toward continuing online learning after the pandemic.
**H6:** During the pandemic, individual learners’ states of online learning have a significant positive effect on online task challenges undertaken after the pandemic.

For the study of flow experience, researchers generally agree on the use of three flow stages as a framework: flow conditions, flow experience, and flow outcomes (Trevino and Webster, 1992; Ghani and Deshpande, 1994; Ghani, 1995). Among the condition factors, Novak (1999) place clear goals, immediate feedback, and the match of challenges with skills as antecedents to the occurrence of mental flow experiences, while Chen et al. (2000) see clear goals, immediate feedback, potential control, and a combination of action and awareness as antecedents to flow. Although the division of antecedents varies, it can be inferred that goals, skills, and challenges are important antecedents, and the achievement of learning goals in the online learning domain must experience the completion of tasks and challenges in the learning process. According to Csikszentmihalyi (1990) definition, the mental flow experience is optimal when challenges and skills are balanced, and this state will promote perceived control. The more motivated the learner is, and the better their self-monitoring of learning time management and task completion, the better the learning process will be to ensure a good experience and thus a high level of satisfaction (Jiang et al., 2017), thereby giving learners a more positive attitude. On this basis, this article proposes the following hypotheses:

**H7:** During the pandemic, learners’ online task challenges have a significant positive effect on their perceived behavioral control of online learning after the pandemic.
**H8:** During the pandemic, learners’ perceived behavioral control of online learning has a significant positive effect on their attitude to continuing online learning after the epidemic.

According to the TPB, attitudes, subjective norms, and perceptual behavioral control all have a significant influence on willingness. This theory is also widely used in the field of online learning. During the pandemic, university students’ home-based learning changed from the traditional offline learning style, and others’ opinions could hardly influence students’ online learning norms. Therefore, from the perspective of psychological, internal factors of university students, this article mainly predicts learners’ willingness to engage in future online learning based on their attitude according to planned behavior theory and perceived behavioral control. As is confirmed by many studies, when learners have a positive attitude toward online learning, their willingness to participate in online learning is higher (Yi and Hwang, 2003; Liu et al., 2009; Park, 2009; Sawang et al., 2014; Valtonen et al., 2015; Chu and Chen, 2016). Perceived behavioral control refers to how learners evaluate their ability and resources and is also a key factor in predicting behavioral intention. Ndubisi (2004) found that perceived behavioral control has a significant positive impact on online learning intention. Zhou (2016) found that learning attitude and perceived behavioral control have a significant impact on students’ willingness to use massive open online course (MOOC) in a study of Chinese university students’ acceptance of MOOC. Therefore, this article takes attitude and perceived behavioral control as the intermediary variables of the three elements of flow experience theory to predict the influence that learners have on their willingness to participate in future online learning. The following hypotheses are proposed:

**H9:** Learners’ attitude toward online learning has a significant positive impact on their willingness to use online learning after the pandemic.
**H10:** Learners’ perceived behavior control of online learning has a significant positive impact on their willingness to use online learning after the pandemic.

## Research Methods

This section is divided into two parts: questionnaire design (see section “Questionnaire Design”) and sample selection and preliminary data statistics description (see section “Sample Selection and Data Statistics”).

### Questionnaire Design

The questionnaire designed for this study mainly includes three parts, namely, research background, respondents’ basic information, and measurement scale of the theoretical model. The scale of the theoretical model refers to the existing research literature and the online learning environment during the pandemic. It adopts a five-point Likert Scale, where 1 means that the question in the questionnaire is totally inconsistent with the respondent’s situation (feeling), while 5 means it is completely consistent. According to Csikszentmihalyi and LeFevre (1989) description of mental flow, this study adopts the questions about concentration raised by Ghani and Deshpande (1994), those about pleasure raised by Moon and Kim (2001), and another question about sense of immediacy. Artifact experience refers to learners’ feelings about the usability and ease of use of artifacts when using the platform, and their feelings about the richness and quality of resources of the online learning platform (Ji, 2013; Li and Xiao, 2019), for which three questions were developed. According to the framework of three flow stages of mental flow (Trevino and Webster, 1992; Ghani and Deshpande, 1994; Ghani, 1995), goals, skills, and challenges are important conditions for the generation of mental flow, for which four questions of task challenges were developed (Jackson and Marsh, 1996; Guo et al., 2016). In the TPB, the attitude was covered by the three questions raised by Thompson et al. (1991), perceived behavior control covered by three questions based on Taylor and Todd (1995) and Zhou (2016), and the willingness to participate in online learning answered by two questions raised by Davis (1989) and Lung-Guang (2019). Appendix 1 lists the complete questions used in this study. To ensure the quality of questionnaire collection, the research group sets a question in the questionnaire to test whether the respondents fill in the questionnaire carefully, which asks them to select option D among the options presented. If the respondents choose any option other than D, the questionnaire was judged as invalid and excluded.

### Sample Selection and Data Statistics

This study took the undergraduate students from 1,243 undergraduate schools and colleges who participated in online learning during the pandemic as the research objects. According to Yemane (1967) formula, the sample size of universities is *n* = 42.91, and the calculated sampling rate is *r* = 3.45% [the calculation formula is shown in Formula (1)].


(1) $$n=N/\left(1+{\mathrm{Ne}}^{2}\right)$$

Here, “*n*” represents the sample size of universities, “*N*” is the number of all Chinese universities, and *e* is the accuracy, set to 15%.

**FIGURE 2:**
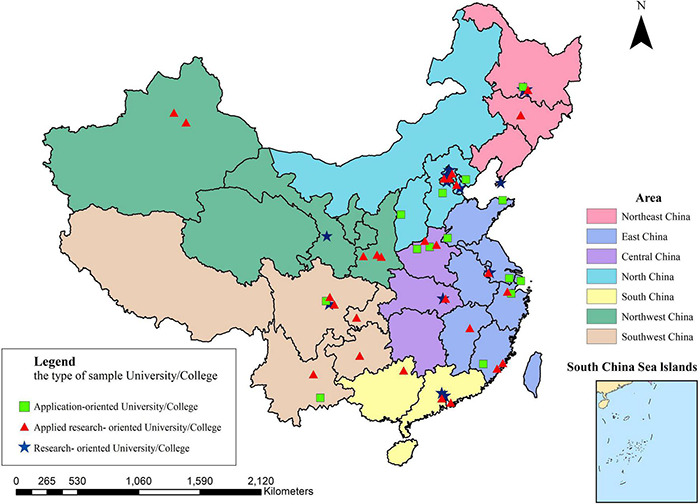
Distribution of questionnaires. Research-oriented University/College is a University/College that offers a comprehensive bachelor’s degree program, puts Research first, and is committed to high-level talent development and scientific research and development. Applied research-oriented University/College is a new type of research University/College which emphasizes more on practical application ability, practical ability, innovation ability, and creativity than academic research University/College. Application-oriented University/College refers to undergraduate institutions of higher learning with application-oriented orientation rather than scientific research orientation, focusing on undergraduate education, as opposed to the concept of academic University/College.

A sample survey was conducted according to the geographical division of Chinese universities (seven regions, namely, Northeast China, North China, East China, South China, Central China, Northwest China, and Southwest China). The research group carried out random sampling for each region whose number was not less than 150%r and conducted a questionnaire survey in universities selected by random sampling. The research group obtained permission from teachers in sampled universities, mainly through telephone or network, and then collected data through a questionnaire that was spread by these teachers. From June 8th to 30th, 2020, the research group picked the sampled universities and collected data. Ultimately, the group received effective responses from 54 universities. Information on the number of universities in each region and their sampling quantity is listed in Table 1. The effective sampling rate of universities in all regions was higher than the calculated sampling rate except that in East China, which is slightly lower than the calculated sampling rate. The distribution of effective sampled universities is given in Figure 2.

**TABLE 1:** Number of universities in different regions and their sampling numbers.

Region	Number of universities	The sample size of 150%r in each region	Number of universities by random sampling	Number of universities with effective response	Effective sampling rate	Comparison of effective sampling rate with nationally calculated sampling rate (*r*)
Northeast China	140	7.25	9	6	4.29%	>3.45%
North China	202	10.46	12	12	5.94%	>3.45%
East China	373	19.31	20	12	3.22%	<3.45%
South China	108	5.59	6	5	4.63%	>3.45%
Central China	173	8.96	9	6	3.47%	>3.45%
Northwest China	107	5.54	8	5	4.67%	>3.45%
Southwest China	140	7.25	8	8	5.71%	>3.45%

Thanks to the active cooperation of teachers in randomly sampled universities, the research group finally collected 6,933 questionnaires and excluded 1,477 questionnaires that failed the validity testing question. In the end, 5,456 valid questionnaires were collected, with an effective rate of 78.64%. Among the respondents, 2,341 were male students, accounting for 57.1%, and 3,115 were female students, accounting for 42.9%. A total of 3,550 respondents majored in science and engineering, accounting for 65.1%, and 1,906 respondents majored in culture and management, accounting for 34.9%. The vast majority were freshmen, sophomores, and juniors, accounting for 93.71%. Among the respondents, 45.1% attended 6 to 8 online learning courses during the pandemic and 40.1% spent an average of 5–6 h on online learning every day during the pandemic. Details of respondents are shown in Table 2.

**TABLE 2:** Profile of respondents.

	Frequency	Percent
***Gender***		
Male	2341	42.91%
Female	3115	57.09%
***Grade***		
Freshman	1930	35.37%
Sophomore	1554	28.48%
Junior	1629	29.86%
Senior	343	6.29%
***Professional category***		
Science and engineering	3550	65.07%
Culture and management	1906	34.93%
***Number of online courses during the pandemic***		
2 and less	173	3.17%
3–5 courses	593	10.87%
6–8 courses	2462	45.12%
9–10 courses	1431	26.23%
11 courses or more	797	14.61%
***Average daily online learning hours duration during the pandemic***		
1 h or less	131	2.40%
1–2 h (including 2 h)	483	8.85%
3–4 h (including 4 h)	1804	33.06%
5–6 h (including 6 h)	2190	40.14%
7 h and above	848	15.54%

## Data Analysis Results

This study applied the partial-least-squares regression analysis tool in evaluating the validity and reliability of the measurement model and quality in testing the hypothesis between structural models and in assessing the significance of load and path coefficient.

### Measurement Model

This study first calculated the value of the Cronbach’s alpha, which was used to test the overall reliability of the questionnaire. Generally, when the Cronbach’s alpha value is greater than 0.9, the result is highly credible; when it is between 0.7 and 0.9, it is very credible (Wang et al., 2017; Zhao et al., 2018). In this study, the Cronbach’s alpha value of the total scale is 0.984, that of each subscale is greater than 0.7, and the compound reliability (CR) of each variable is greater than 0.7 (Hair et al., 2009). This implies that the scale has high reliability, good stability, and internal consistency. In terms of convergence validity, the factor loading of each variable is greater than 0.7, and the average variance of each variable is also greater than 0.5 (Hair et al., 2014), indicating that the scale has a good convergence effect (Table 3). From the perspective of discrimination validity (Table 4), the correlation coefficients among all variables are less than the square root of AVE, indicating that the scale has good discrimination validity (Fornell and Larcker, 1981; Chin, 1998).

**TABLE 3:** Factor loading, Cronbach’s α, CR, and AVE of constructs.

Variable	Item	Loadings	Cronbach’s alpha	AVE	CR
Person (Online learning state of respondents)	P1	0.846	0.789	0.703	0.877
	P2	0.856			
	P3	0.813			
Artifact (Online learning platforms and resources)	A1	0.883	0.803	0.717	0.884
	A2	0.817			
	A3	0.839			
Task (Online learning task)	T1	0.847	0.864	0.711	0.908
	T2	0.877			
	T3	0.813			
	T4	0.834			
Perceived Behavioral Control	PBC1	0.864	0.792	0.705	0.878
	PBC2	0.828			
	PBC3	0.826			
Attitude	ATT1	0.800	0.842	0.761	0.905
	ATT2	0.902			
	ATT3	0.911			
Intention	INT1	0.958	0.909	0.917	0.917
	INT2	0.957			

**TABLE 4:** Simple correlation matrix and discriminatory validity.

Construct	Task	Artifact	Person	Attitude	Intention	Perceived behavioral control
Task	**0.843**					
Artifact	0.692	**0.847**				
Person	0.765	0.665	**0.839**			
Attitude	0.581	0.588	0.619	**0.872**		
Intention	0.572	0.59	0.596	0.776	**0.958**	
Perceived behavioral control	0.659	0.714	0.595	0.584	0.575	**0.840**

*Bold means square roots of AVE values.*

### Structural Model

**FIGURE 3:**
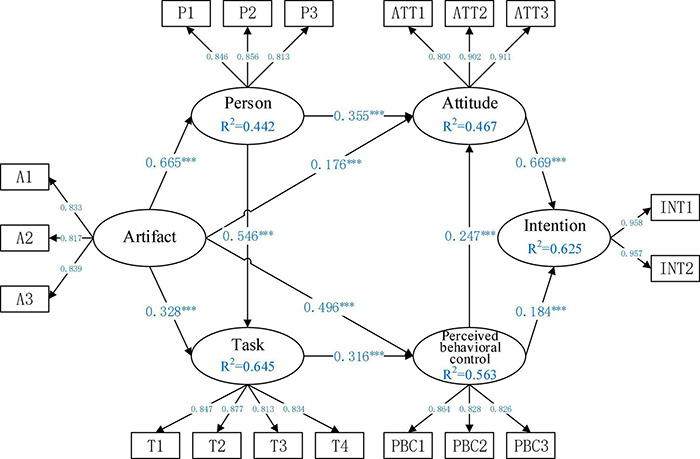
Structural model and paths coefficient. ***refer to the significance level at 0.01.

This study repeatedly sampled 5,456 cases using the bootstrapping function of SmartPLS 3.0 software to verify the relationships between model variables. Figure 3 shows all the path coefficients of the model and the variance of interpretation. The data show that the highest *R*^2^ value of the task is 0.645. This means that the artifact and person have the greatest influence on the task of Chinese undergraduates’ online learning during the pandemic, as their interpretation variance reached 64.5%. In descending order of interpretation variance, the influence of attitude and perceived behavioral control on intention ranks second at *R*^2^ = 0.62. For the influence of artifact and task on perceived behavioral control, *R*^2^ = 0.563; for the influence of artifact and perceived behavioral control on attitude, *R*^2^ = 0.467; finally, for the influence of artifact on the person, *R*^2^ = 0.442. The Standardized Root Mean Square Residual (SRMR) of the model is 0.065 (less than 0.08), which indicates that the model fits well (Hu and Bentler, 1998). The test results of the research hypothesis are shown in Table 5, and the significance of each path reached the level of *P* < 0.001, indicating that the research hypothesis is well supported.

**TABLE 5:** The *t*-value of research hypotheses and path coefficients.

Hypothesis	Relationship	Path coefficient	*t*-value	*p*-value	Conclusion
H1	Artifact–Person	0.665	74.022	***	Support
H2	Artifact–Task	0.328	24.032	***	Support
H3	Artifact–Attitude	0.176	9.658	***	Support
H4	Artifact–Perceived Behavioral Control	0.496	33.538	***	Support
H5	Person–Attitude	0.355	22.342	***	Support
H6	Person–Task	0.546	42.325	***	Support
H7	Task–Perceived Behavioral Control	0.316	20.672	***	Support
H8	Perceived Behavioral Control–Attitude	0.247	14.471	***	Support
H9	Attitude–Intention	0.669	62.682	***	Support
H10	Perceived Behavioral Control–Intention	0.184	15.242	***	Support

***refer to the significance level at 0.01.

## Discussion and Implications

During the pandemic, students in Chinese universities have had intensive access to various online learning platforms through online learning. As learning delivery has shifted from onsite to online, university students have experienced massive online learning activities. Online learning, like other forms of learning, is an experience wrought with emotions (Sharpe and Benfield, 2005). Learners may feel depressed, desperate, happy, warm, lonely, or have a sense of belonging (Xiao, 2020). This study reveals the mental flow experience (artifact experience, respondent’s online learning state, and online learning tasks and challenges) of online learning of Chinese undergraduates during the pandemic. The research results are generally consistent with those of Davis and Wong (2007), Lee (2010), and Wang et al. (2017). Therefore, even in the case of a sudden outbreak, flow experience and TPB still have their applicability.

According to the PAT model (Finneran and Zhang, 2003), the artifact is one of the conditions for creating mental flow. In the context of this pandemic, both large-scale online learning and artifact experience (learning platform and resources) have had a positive impact on the process and results of online learning. This study shows that the online learning artifact experience has had significant and positive impacts on the individual state of Chinese undergraduates during the pandemic (*R* = 0.665, *P* = 0.000, H1) and also actively promoted their online learning tasks (*R* = 0.328, *P* = 0.000, H2). According to Norman (1988), a good artifact experience enables learners to ignore the artifact itself and focus on the task, thus promoting learners’ task attainment. At the same time, the artifact experience of online learning has also significantly and positively influenced the perceived behavioral control of Chinese undergraduates during the pandemic (*R* = 0.496, *P* = 0.000, H4). Through learning platform artifacts, learners become familiar with the functions of the online learning platform, which promotes their control ability of these platforms and is conducive to improving their ability to control the perceived behavior of online learning in future blending learning. According to the empirical results, the positive artifact experience of online learning during the pandemic has significantly and positively influenced students’ attitudes toward further online learning (*R* = 0.176, *P* = 0.000, H3). The better the experience of the online learning platform and learning resources, the more students found online learning interesting and the more inclined they were to choose online learning in future learning. This study describes the artifact experience according to the aspects of interactivity (Chen and Jia, 2020), usefulness, resource quality, and richness (Lee, 2010), which constitute important links for learners in using the platform. On the whole, with a good artifact experience, students can significantly improve their learning state (better concentration and happier learning), and be actively driven to complete tasks and challenges. Besides, a good artifact experience also has a positive role in promoting the further use of perceived behavior control and learning attitude in online learning.

For Chinese undergraduates, the individual’s psychological state has a significant positive impact on completing tasks and challenges in online learning (*R* = 0.546, *P* = 0.000, H6) and on the attitude to further adopting online learning (*R* = 0.355, *P* = 0.000, H5). This is consistent with the research of Richard and Chandra (2005) and Sánchez-Franco (2007). The research of Gong et al. (2016) finds that when students experience positive emotions during engagement with blending learning, they accomplish online learning tasks with better performance and higher satisfaction. These results show that learners’ individual learning state affects their perception of tasks and challenges. Similarly, a positive state during online learning can both improve their ability to cope with tasks and challenges in learning and also effectively facilitate further adoption of online learning. During the pandemic, students’ positive experiences of tasks and challenges in online learning significantly improved their perceived behavior control in online learning (*R* = 0.316, *P* = 0.000, H7). When students realize that they have clear goals, they can control learning progress and learning outcomes, satisfaction with online learning is effectively enhanced (Jiang et al., 2017), the subjective initiative in using the online learning platform and learning resources can be improved, ability to solve problems in the face of difficulties can be strengthened, and they will have greater confidence and greater control of their perception and behavior in further adopting online learning platforms and resources (Jiang et al., 2017).

Consistent with most research results, this study concludes that Chinese undergraduates’ perceived behavior control in further adopting online learning has a significant positive impact on their attitude (*R* = 0.247, *P* = 0.000, H8) and intention to further adopt online learning (*R* = 0.184, *P* = 0.000, H10) (Moon and Kim, 2001; Zhou, 2016). Through the online learning experience during the pandemic, learners’ positive attitude (interest and likeness) to further adopt online learning can significantly enhance their intention to further adopt online learning (*R* = 0.669, *P* = 0.000), which is consistent with the research results of Park (2009), Sawang et al. (2014), and Valtonen et al. (2015).

In the context of COVID-19 pandemic, this study provides a new perspective for a wide range of online teaching and learning. It also proves the wide applicability of flow experience and TPB. However, this study only considered the core elements of flow experience and the TPB, in fact, there may be more key influencing factors, such as the impact of COVID-19 itself was not considered. This lays a foundation for further research.

## Conclusion

Due to the long- and large-scale online education during the pandemic, Chinese higher education is experiencing dramatic changes and this would result in blended learning in the postpandemic era. This study aims to provide theoretical and practical support for a better design of a blended learning model in Chinese higher education institutions in the postpandemic era. The influence of the online learning experience of university undergraduates on their willingness to continue with online learning in the context of COVID-19 pandemic is explored to help shape postpandemic education. This is the first study on the recent reforms in teaching and learning in Chinese higher education in the postpandemic era from a large scale.

To understand the past experience on future intentions for Chinese students to continue online learning, this article integrates the theory of mental flow experience and the TPB, to measure the learning experience of online learners during COVID-19 pandemic and predict the impact on learners’ planned future online learning behavior. This research results in a theoretical basis for the reform of undergraduate education in Chinese universities in the postpandemic era.

The results show that during the pandemic, factors such as learners’ personal learning state, online learning artifacts, and online learning task and challenges all have positive impacts on learners’ perceived behavior control and attitude in further adopting online learning, thus actively promoting their willingness to engage in future online learning. Therefore, to promote educational reform toward a blended learning model in Chinese universities in the postpandemic era, this study offers the following specific recommendations regarding online learning platform design, curriculum task design, and learners’ learning state.

Learners always perceive online learning as a lonely experience (Simpson, 2014). Therefore, it is important to consider how to fully exploit the unique advantages of online learning artifacts, improve the emotional presence of online learning, enhance the individual learning state of learners, and formulate scientific and reasonable online learning tasks and schedules for the good experience of online learning (Cleveland-Innes and Campbell, 2012). In this way, students could gain pleasure, eliminate their loneliness, and improve the learning effect during online learning. First, as for artifacts, online learning platforms should provide functions able to fully meet the learning needs of learners and provide reliable quality and convenient access to learning resources. In terms of interactivity, there are three types of interaction among online participants, namely, teacher–learner, learner–content, and learner–learner (Moore, 1989). Effective interaction in online learning significantly improves online learners’ learning attitude, learning satisfaction, and willingness to continue to adopt online learning (Hrastinski, 2009), so online courses should effectively make use of four-dimensional interactions between learner, teacher, resource, and learning. Second, as is indicated by the significant positive influence of individual learning state on willingness to engage in online learning, the proposals and developers of online learning should beware of making online learning a burden and solely a form of learning. Instead, they should enable learners to fully feel the pleasure of being immersed in it. For example, some researchers find that colorful and personalized learning content in a multimedia environment could successfully trigger students’ positive emotions and improve their academic performance (Plass et al., 2014). Meanwhile, online learners also need to actively organize and adjust their learning state to improve the learning effect. Finally, as for the design of curriculum tasks, it is necessary to give learners clear goals and to consider students’ levels so that curriculum challenges accurately match students’ abilities. The task requirements should conform to students’ cognitive ability, be able to facilitate students to accomplish tasks, and stimulate their internal motivation for online learning (Joo et al., 2013). For example, by associating tasks with students’ real lives and future careers, students can realize the importance and value of learning tasks (Gong et al., 2016), after all, only a good mental flow experience can provide strong support for the normalized development of online learning.

## Data Availability Statement

The original contributions presented in the study are included in the article/supplementary material, further inquiries can be directed to the corresponding author/s.

## Ethics Statement

Ethical review and approval was not required for the study on human participants in accordance with the local legislation and institutional requirements. Written informed consent from the participants’ legal guardian/next of kin was not required to participate in this study in accordance with the national legislation and the institutional requirements.

## Author Contributions

YA and LZ: research design and methodology. LZ and YW: data collection and analysis. YW, YA, and TW: draft and editing. All authors: contributed to the article and approved the submitted version.

## Conflict of Interest

The authors declare that the research was conducted in the absence of any commercial or financial relationships that could be construed as a potential conflict of interest.

## Publisher’s Note

All claims expressed in this article are solely those of the authors and do not necessarily represent those of their affiliated organizations, or those of the publisher, the editors and the reviewers. Any product that may be evaluated in this article, or claim that may be made by its manufacturer, is not guaranteed or endorsed by the publisher.

## Appendix

**Appendix:** Survey items.

Constructs	No	Items	References
Artifact	A1	The rich online learning platforms and their functions during the pandemic can meet the needs of my online learning.	Hoffman and Novak, 1996; Finneran and Zhang, 2003
	A2	In the online study during the pandemic, I can interact effectively with teachers and classmates through the platform.	
	A3	There are rich resources (audio, graphics, and text) in online learning during the pandemic, which can satisfy my demands for learning resources.	
Task	T1	When I start online learning during the pandemic, I have clear learning goals.	Ghani, 1995; Finneran and Zhang, 2003; Guo et al., 2016
	T2	When pursuing online during the pandemic, I have strong time management ability and I’m able to control study progress.	
	T3	When pursuing online during the pandemic, I am able to complete the course tasks of online learning.	
	T4	During the pandemic, I learn online and achieve the same effect as the previous offline learning.	
Person	P1	When learning online during the pandemic, I can concentrate well.	Csikszentmihalyi and LeFevre, 1989; Ghani and Deshpande, 1994; Moon and Kim, 2001
	P2	I feel very happy in online learning during the pandemic.	
	P3	When pursuing online during the pandemic, I have the same feeling as offline learning in the past.	
Perceived behavioral control	PBC1	I am equipped with the skills and knowledge necessary for online learning.	Ajzen, 1991; Taylor and Todd, 1995; Davis and Wong, 2007
	PBC2	I am familiar with the functions of various online learning platforms and can operate them skillfully.	
	PBC3	With the online learning experience during the pandemic, I find it easy to use the online learning platform.	
Attitude	ATT1	I use online learning during the pandemic, and it will difficult for me to stop using it now and in the future.	Thompson et al., 1991; Zhou, 2016
	ATT2	I think online learning is a more interesting way of learning than offline learning.	
	ATT3	I like learning online.	
Intention	INT1	After the pandemic, I am willing to continue to use online learning.	Davis, 1989; Lung-Guang, 2019
	INT2	I am willing to stick to online learning in the future.	
